# High hydrostatic pressure (30 atm) enhances the apoptosis and inhibits the proteoglycan synthesis and extracellular matrix level of human nucleus pulposus cells via promoting the Wnt/β-catenin pathway

**DOI:** 10.1080/21655979.2022.2025518

**Published:** 2022-01-31

**Authors:** Zongting Shi, Jun He, Jian He, Yuan Xu

**Affiliations:** a Department of Spine, Beijing University of Chinese Medicine Third Affiliated Hospital, Beijing, China; b Department of Orthopedics, Zhejiang Hospital, Hangzhou City, Zhejiang Province, China

**Keywords:** Hydrostatic pressure, human nucleus pulposus cells, apoptosis, viability, Wnt/β-catenin pathway

## Abstract

Hydrostatic pressure is known to regulate bovine nucleus pulposus cell metabolism, but its mechanism in human nucleus pulposus cells (HNPCs) remains obscure, which attracts our attention and becomes the focus in this study. Specifically, HNPCs were treated with SKL2001 (an agonist in the Wnt/β-catenin pathway) or XAV-939 (an inhibitor of the Wnt/β-catenin pathway), and pressurized under the hydrostatic pressure of 1, 3 and 30 atm. The viability, apoptosis and proteoglycan synthesis of treated HNPC were assessed by CCK-8, flow cytometry and radioisotope incorporation assays. The levels of extracellular matrix, Collagen-II, matrix metalloproteinase 3 (MMP3), Wnt-3a and β-catenin were measured by toluidine blue staining, immunocytochemistry and Western blot. Appropriate hydrostatic stimulation (3 atm) enhanced the viability and proteoglycan synthesis yet inhibited the apoptosis of HNPCs, which also up-regulated extracellular matrix and Collagen-II levels, and down-regulated MMP3, Wnt-3a and β-catenin levels in treated HNPCs. Furthermore, high hydrostatic pressure (30 atm) inhibited the viability and proteoglycan synthesis, and promoted the morphological change and apoptosis of HNPCs, which also down-regulated extracellular matrix and Collagen-II levels and up-regulated MMP3, Wnt-3a and β-catenin levels. Besides, SKL2001 reversed the effects of hydrostatic pressure (3 atm) on inhibiting Wnt-3a, β-catenin, and MMP3 levels and promoting Collagen-II level in HNPC; whereas, XAV-939 reversed the effects of high hydrostatic pressure (30 atm) on promoting MMP3, Wnt-3a, and β-catenin levels and inhibiting Collagen-II level and proteoglycan synthesis of HNPCs. Collectively, high hydrostatic pressure promoted the apoptosis and inhibited the viability of HNPCs via activating the Wnt/β-catenin pathway.

## Graphical abstract

**Figure:**
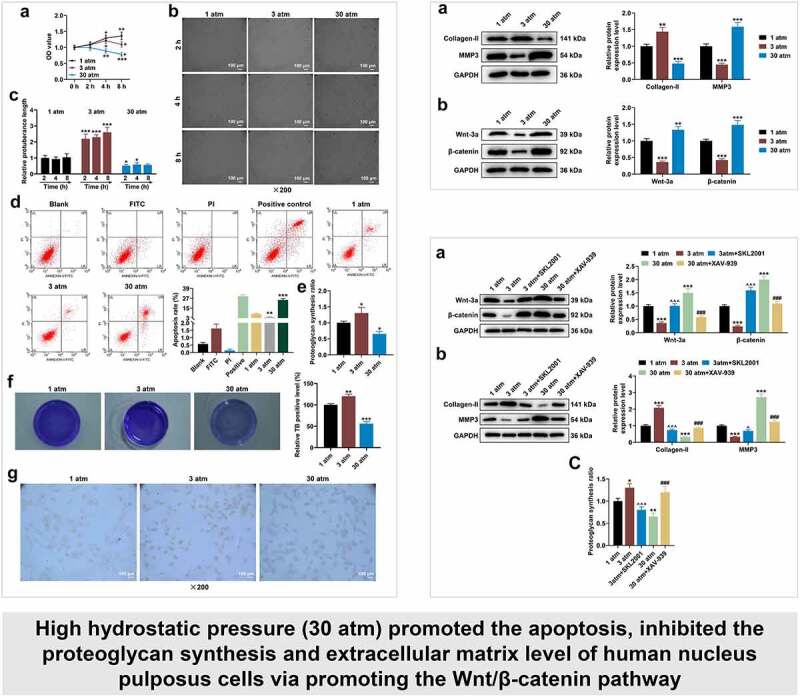

## Introduction

Low back pain is a common musculoskeletal symptom, and data from related surveys mirror that; in the United States, approximately 25% of people suffer from neck and back pain [1]. In recent years, more and more researches have revealed that intervertebral disc degeneration is one of the main causes of low back pain [2,3], posing challenges to orthopedic clinical treatment mainly consisting of medication, physiotherapy and surgery [4]. Although surgical treatment relieves clinical symptoms to some extent, the therapeutic effect is unsatisfactory owing to the dearth of comprehension on the exact regulatory mechanism of intervertebral disc degeneration [5,6]. Therefore, exploring the pathogenesis of disc degeneration is helpful to provide a reliable reference for the treatment of lumbar disc herniation [7].

At present, the pathogenesis of intervertebral disc degeneration is more and more deeply studied, the biomechanics theory has been used as the most classical and intuitive explanation of this disease in recent decades [8]. The intervertebral disc is mainly composed of three parts: nucleus pulposus, annulus fibrosus and cartilaginous endplate [4]. Relevant studies have indicated that in daily life, a wide range of impacts and repetitive loading in the skeletal system are important predisposing factors for intervertebral disc degeneration [8]. Hydrostatic pressure is a mechanical stimulus [9]. During joint loading, fluid is expelled from the tissue, friction between the fluid and the solid phase of the tissue dissipates energy from the applied load; in the joint, cartilage is typically exposed to stresses between 0.2 and 10 MPa and these stresses are converted to hydrostatic pressure due to fluid phase pressurization [10]. Hydrostatic pressure has a profound effect on cartilage metabolism, especially in weight-bearing areas of the skeletal system [11,12]. To the best of our knowledge, 3 atm of hydrostatic pressure is equivalent to the pressure in the human lumbar intervertebral disc in the resting position, and 30 of atm hydrostatic pressure is tantamount to the simultaneous forward leaning of a person while lifting weights [13].

Intervertebral disc degeneration is characterized by the decrease of nucleus pulposus cells [14]. Relevant findings have proved that protecting nucleus pulposus cell from death is beneficial for improving intervertebral disc degeneration *in vivo* [15]. In addition, a study has pointed out that dynamic hydrostatic loading regulates and appropriate hydrostatic stimulation contributes to nucleus pulposus cell metabolism. Further, long-term improper and excessive pressure will lead to nucleus pulposus apoptosis and cause intervertebral disc degeneration, leading to the occurrence of low back pain [16]. Besides, hydrostatic pressure affects the proteoglycan synthesis and the level of collagen in the intervertebral disc cells [17,18]. However, the underlying mechanism of hydrostatic pressure impacting human nucleus pulposus cells (HNPCs) remains unclear.

Previous results have demonstrated that hydrostatic pressure regulates oxidative stress and chondrocyte metabolism in human osteoarthritic chondrocytes by regulating microRNA-mediated Wnt/β-catenin signaling pathway [19]. Additionally, a strong relationship between the progress of intervertebral disc degeneration and Wnt/β-catenin signaling pathway has been reported in the literature [20]. Interestingly, silencing taurine up-regulated 1 protects HNPCs from tumor necrosis factor-α-induced apoptosis, and also enhances cell proliferation by blocking Wnt/β-catenin pathway in HNPCs [20,21]. However, the relationship between the hydrostatic pressure and Wnt/β-catenin signaling pathway in HNPCs awaits to be investigated.

We hypothesized that hydrostatic pressure regulates HNPCs metabolism via regulating the Wnt/β-catenin signaling pathway. The goal of this research was to fathom out the effect and underlying mechanism of hydrostatic pressure in HNPCs and whether the Wnt/β-catenin pathway is involved in its regulatory mechanism, so as to provide a theoretical basis for the clinical treatment of intervertebral disc degeneration.

## Materials and methods

### Cell culture

HNPCs (4800, ScienCell Research Laboratories, Carlsbad, California, USA) were purchased. For the cell culture, the incubator (HH.CP-01) obtained from Grows Instrument Co., Ltd (Shanghai, China) was used to culture cells. Then, HNPCs were cultured in Nucleus Pulposus Cell Medium (4801, ScienCell Research Laboratories, Carlsbad, California, USA) at 37°C with 5% CO_2_, and the cell medium was replaced 2–3 times a week.

### Cell treatment

In this study, the hydrostatic pressure system was structured by Beijing Shiji Senlang Experimental Instrument Co., Ltd. (Beijing, China). The HNPCs were cultivated *in vitro*, and the cell suspension was prepared through trypsin (C0205, Beyotime, Shanghai, China) digestion when the HNPC confluence reached 90%. As previously described, HNPCs were collected, charged with cell medium in a plastic syringe (A2292, Aladdin, Shanghai, China), and placed in the water-filled hydrostatic pressure-controlled vessel. Then, cells were pressurized for 2, 4 and 8 hours (h) under the hydrostatic pressure of 1, 3 and 30 atm (atm = atmospheres) [22]. Afterward, HNPCs were collected for subsequent studies.

In another test, an agonist (SKL2001, R2033064) and an inhibitor (XAV-939, R2033040) of the Wnt/β-catenin signaling pathway were purchased from Shanghai Rechemscience Co., Ltd. (Shanghai, China). In accordance with the previous description [23,24], HNPCs were cultured in the cell medium supplemented with SKL2001 (40 μM) or XAV-939 (10 μM), and then cells were transferred using a plastic syringe to the water-filled hydrostatic pressure-controlled vessel. After that, cells were pressurized under the hydrostatic pressure of 3 or 30 atm for 2 h, and then collected for subsequent researches.

### Cell viability assay

The viability of treated HNPCs was assessed using Cell Counting Kit-8 (CCK-8) assay [25]. In this detection, the CCK-8 (AC11L054) was ordered from Shanghai LIFE iLAB Technology CO., LTD (Shanghai, China). Subsequently, the HNPCs were pressurized for 2, 4 and 8 h under the hydrostatic pressure of 1, 3 and 30 atm, followed by another 2-h culture in an incubator with the treatment of 10 μL of CCK-8 solution. Finally, the absorbance (at 450 nm) was determined using the microplate reader (ELx808, BioTek, Winooski, Vermont, USA).

### Observation of cell morphology

The cell morphology of HNPCs was observed using microscope [15]. HNPCs were treated and removed using the plastic syringe. Afterward, the treated cells were washed with Phosphate Buffered Saline (PBS; P274233, Aladdin, Shanghai, China), and then observed (under 200 × magnification) using a light microscope (DMD108, Leica, Wetzlar, Frankfurt, Germany).

### Flow cytometric analysis of apoptosis

The apoptosis of treated HNPCs was evaluated using flow cytometry [26]. Prior to this evaluation, the Annexin V-FITC/Propidium Iodide Apoptosis Detection Kit (C1062M) was purchased from Beyotime (Shanghai, China), and treated HNPCs were collected and digested with Trypsin solution. Briefly, treated HNPCs were resuspended with PBS and the cell concentration was adjusted, after which cells (5 × 10^4^) were centrifuged (1000 × *g*) using a centrifuge (E2615, Beyotime, Shanghai, China) for 5 min. Afterward, cells were added with 195 μL AnnexinV–FITC conjugated solution, 5 μL of Annexin V-FITC solution and 10 μL of Propidium Iodide solution, followed by 15-min incubation at room temperature in the dark. Ultimately, the apoptosis of treated HNPCs was analyzed using flow cytometer (CytoFLEX, Beckman Coulter, Inc., Kraemer Boulevard Brea, California, USA) and the Kaluza C software (v. 1.1.2, Beckman Coulter, Indianapolis, Indiana, USA).

### Measurement of proteoglycan synthesis

Following previous works [27], the proteoglycan synthesis rate of HNPCs was evaluated using radioisotope incorporation assay. The treated cells were cultured in cell medium containing 185 kBq/mL of ^35^S-sulfate (Amersham, Los Angeles, California, USA) at 4°C for 1 h in order to allow uniform and complete diffusion of ^35^S-sulfate into the cells. After that, cells were treated according to the experimental protocol, and frozen at −20°C to terminate proteoglycan synthesis. To determine proteoglycan synthesis, cells and culture medium were thawed and dissolved, cell samples and culture medium were loaded together in the cellulose dialysis bag (cutoff value, 12,000–14,000 MW, FY24844, FEIYUBIO, Nantong, China), and samples were washed under running water for 3 d until ^35^S-sulfate was undetectable. The radioactivity of samples was measured by a liquid scintillation counter (FB03-000262, Aloka, Tokyo, Japan), and the proteoglycan synthesis rate was calculated according to the Bayliss method [28].

### Toluidine blue staining

The level of extracellular matrix was observed by toluidine blue staining [29]. HNPCs were treated in line with the experimental instructions and then taken out from the plastic syringe. Next, cells were fixed with 95% of ethanol (E111991, Aladdin, Shanghai, China) for 15 seconds (s), stained with toluidine blue solution (G3660, Solarbio, Beijing, China) at room temperature for 5 min, and rinsed in water for 15 min. Finally, the cells were observed using a camera (COOLPIX P1000, Nikon, Tokyo, Japan).

### Immunocytochemistry (ICC)

In the light of a previous delineation, the expression of Collagen-II was observed through ICC [30]. The treated HNPCs were collected and washed with PBS, and then fixed with 4% paraformaldehyde at room temperature for 15 min. Next, cells were treated with 0.2% of Triton X-100 (P0096, Beyotime, Shanghai, China) for 10 min, and incubated firstly with 10% goat serum (C0265, Beyotime, Shanghai, China) for 20 min, and then with the diluted primary antibody (Collagen II Monoclonal Antibody; MA1-37,493, Thermo Fisher Scientific, Waltham, Massachusetts, USA) at 4°C overnight. Subsequently, cells were incubated with the diluted secondary antibody (Rabbit Anti-Mouse IgG H&L; ab6728, Abcam, Cambridge, UK) at room temperature for 1 h. Later, the cells were treated with DAB working solution (P0202, Beyotime, Shanghai, China), and then washed with distilled water. Lastly, the Collagen-II content of cells was observed (under 200 × magnification) using fluorescence microscope (N-STORM, Nikon, Tokyo, Japan).

### Western blot

In this assay, Western blot was performed as previously described [28]. Prior to Western blot assay, the treated cells were harvested (5 × 10^6^) and washed with PBS. Afterward, total proteins were extracted from the treated cells with the help of total protein extraction kit (C1396, Jining Shiye, Shanghai, China), the concentration of which was quantitated uing the bicinchoninic acid (BCA) protein assay kit (C1397, Jining Shiye, Shanghai, China). Later, equal amounts (20 μ) of protein samples were electrophoresed by the SDS-PAGE gel (BB-3702, BestBio, Nanjing, China). Subsequently, the separated proteins were transferred onto the polyvinylidene fluoride (PVDF) membrane (LC2002, Thermo Fisher Scientific, Waltham, Massachusetts, USA), which was blocked with Western Blocking Buffer (BB-3512, BestBio, Nanjing, China) at room temperature for 1 h. After washing with Western Wash Buffer (P0023C3, Beyotime, Shanghai, China), the membrane was incubated with the diluted primary antibody solution at 4°C overnight. Next, the membrane was rinsed in Western Wash Buffer and incubated with secondary antibodies at room temperature for 1 h. Lastly, the membrane was visualized by enhanced chemiluminescence (ECL) working solution (32,209, Thermo Fisher Scientific, Waltham, Massachusetts, USA), and the results were analyzed under the Western blot imaging system (FluorChem M, Alpha Innotech, San Francisco, California, USA). In this research, all information of antibodies is listed in Table 1.

**Table 1.** All antibodies information and sources in Western blot in this study

ID	Catalog number	Company (country)	Molecular weight	Dilution ratio
Wnt-3a	ab81614	Abcam (Cambridge, UK)	39 kDa	1/1000
β-catenin	ab32572	Abcam (Cambridge, UK)	92 kDa	1/1000
Collagen-II	ab188570	Abcam (Cambridge, UK)	141 kDa	1/1000
MMP3	ab53015	Abcam (Cambridge, UK)	54 kDa	1/1000
GAPDH	ab181602	Abcam (Cambridge, UK)	36 kDa	1/10,000
Mouse IgG	ab6789	Abcam (Cambridge, UK)		1/5000
Rabbit IgG	ab205718	Abcam (Cambridge, UK)		1/5000

### Statistical analysis

Statistical analyses were completed using SPSS 20.0. Measurement data were expressed as mean ± standard deviation, which were indicative of three independent experiments. Multiple groups were compared using one-way analysis of variance (ANOVA), followed by Tukey’s post hoc test. *P* < 0.05 was considered as statistically significant.

## Results

We surmised that hydrostatic pressure modulated HNPCs metabolism via regulating the Wnt/β-catenin signaling pathway. This research focused on evaluating the effect and underlying mechanism of hydrostatic pressure in HNPCs and whether the Wnt/β-catenin pathway is involved in its regulatory mechanism. HNPCs were treated with or without SKL2001 and XAV-939, and pressurized under the hydrostatic pressure of 1, 3 and 30 atm. The viability, apoptosis and proteoglycan synthesis of treated HNPCs were assessed by CCK-8, flow cytometer and radioisotope incorporation assays, respectively. The cell morphology was observed using light microscope. The levels of extracellular matrix, Collagen-II, matrix metalloproteinase 3 (MMP3), Wnt-3a and β-catenin were measured by toluidine blue staining, immunocytochemistry and Western blot.

### High hydrostatic pressure (30 atm) inhibited the viability and promoted the morphological change and apoptosis of HNPCs

**Figure 1.**
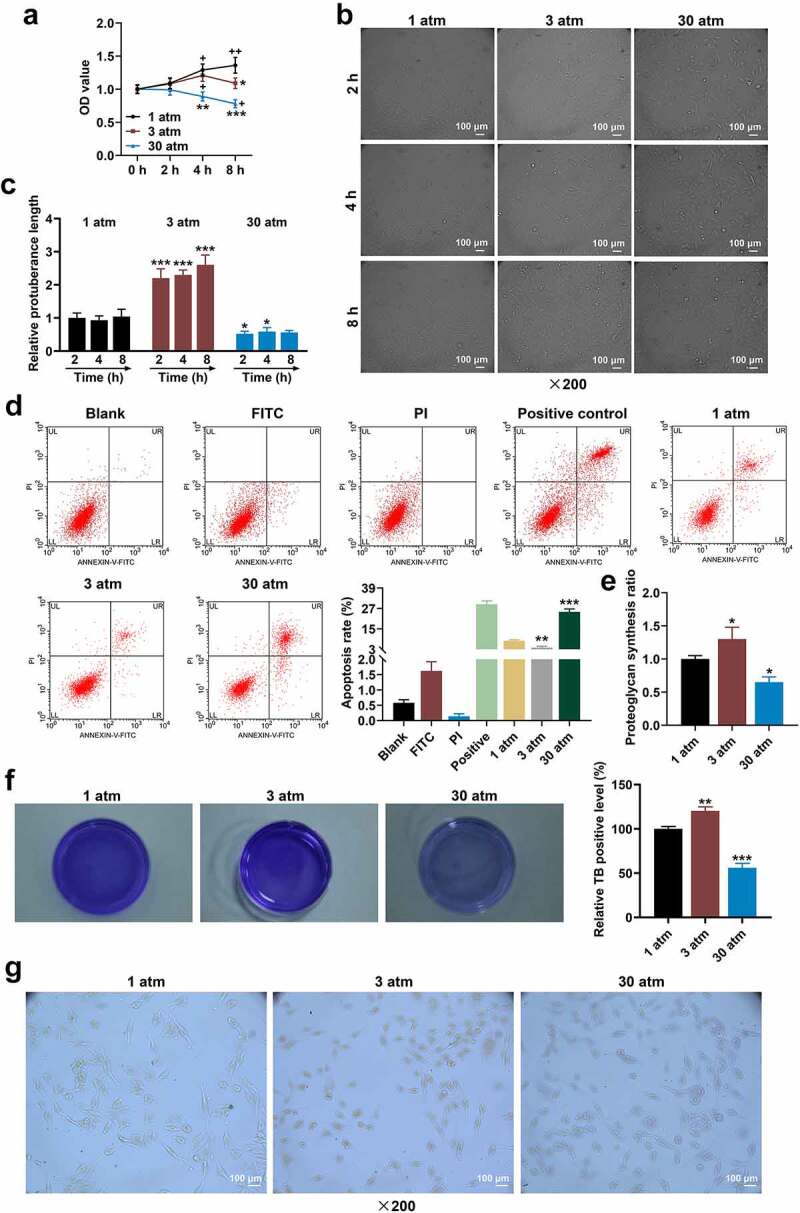
**High hydrostatic pressure (30 atm) inhibited the viability yet promoted the morphological change and apoptosis of HNPCs, and suppressed the proteoglycan synthesis, extracellular matrix level and Collagen-II expression in HNPCs**. (A–G) HNPCs were collected, charged with cell medium in a plastic syringe and placed in the water-filled hydrostatic pressure-controlled vessel. Then cells were pressurized for 2, 4 and 8 hours (h) under the hydrostatic pressure of 1, 3 and 30 atm. Next, the viability of treated HNPCs was assessed using CCK-8 assay (A). The morphology of treated HNPC was observed (under 200 × magnification, Scale bar = 100 μm) using a light microscope (B–C). The apoptosis of treated HNPC was detected using a flow cytometer, and the results were exhibited using the Kaluza C software (D). The proteoglycan synthesis rate of HNPC was evaluated via radioisotope incorporation assay (E). The cellular matrix level (F) and Collagen-II expression (G) in treated HNPC were measured using toluidine blue staining and immunocytochemistry (under 200 × magnification, Scale bar = 100 μm). *P < 0.05, **P < 0.01, ***P < 0.001 vs. 1 atm; ^+^*P* < 0.05, ^++^*P* < 0.01 vs. 0 h. (HNPCs: human nucleus pulposus cells, atm: atmospheres).

Figure 1A reflects the effect of hydrostatic pressure on the viability of HNPCs. After 1  atm hydrostatic pressure treatment, HNPC viability tended to get stronger, which was more evident after 8-h treatment (Figure 1A, P < 0.05). After 3 atm hydrostatic pressure treatment, the viability of HNPCs was first strengthened and then weakened with increasing time, with the highest viability being observed at 4 h. Thirty  atmospheres hydrostatic pressure treatment resulted in gradually decreased HNPCs viability with increasing time, when compared to 1 atm hydrostatic pressure (Figure 1A, P < 0.05). Next, the morphology of treated HNPCs was observed (Figure 1B). 1 atm hydrostatic pressure treatment barely affected morphology of treated HNPCs, while 3 and 30 atm hydrostatic pressure treatments induced the morphological change of HNPCs. Besides, since HNPCs morphology had no difference in different treatment times under the same pressure environment, 2 h was selected as the treatment time for subsequent experiments (Figure 1C, P < 0.05). In addition, 3 atm hydrostatic pressure treatment blocked the apoptosis, but 30 atm hydrostatic pressure treatment markedly accelerated the apoptosis of HNPC, when compared with 1 atm hydrostatic pressure treatment (Figure 1D, P < 0.01). These data manifested that excessive hydrostatic pressure (30 atm) inhibited the viability and promoted the morphological change and apoptosis of HNPCs.

### High hydrostatic pressure (30 atm) diminished the proteoglycan synthesis ratio, extracellular matrix level and Collagen-II expression in HNPCs

A previous study has demonstrated that hydrostatic pressure (3 atm) stimulated the production of metalloproteinases-1 and proteoglycan synthesis [31]. In contrast, high hydrostatic pressure decreased the level of Collagen-II [32]. In this work, the proteoglycan synthesis and cellular matrix level in treated HNPCs were evaluated, uncovering that 3 atm hydrostatic pressure treatment augmented proteoglycan synthesis ratio, while excessive hydrostatic pressure (30 atm) markedly lessened the ratio as compared with 1 atm hydrostatic pressure treatment (Figure 1E, P < 0.05). On the basis of Figure 1F–G, the cellular matrix level and Collagen-II expression (Collagen-II was brownish-yellow after immunostaining) in treated HNPCs were assessed using toluidine blue staining (Figure 1F) and ICC (Figure 1G), with the findings exhibiting that 3 atm hydrostatic pressure treatment promoted the levels of cellular matrix and Collagen-II in HNPCs, but excessive hydrostatic pressure (30 atm) generated the opposite effect. These results implied that high hydrostatic pressure (30 atm) inhibited the viability and promoted the apoptosis of HNPC by suppressing the proteoglycan synthesis, cellular matrix level and Collagen-II expression in HNPCs.

### High hydrostatic pressure (30 atm) down-regulated Collagen-II expression, while up-regulating the levels of MMP3, Wnt-3a and β-catenin in HNPCs

**Figure 2.**
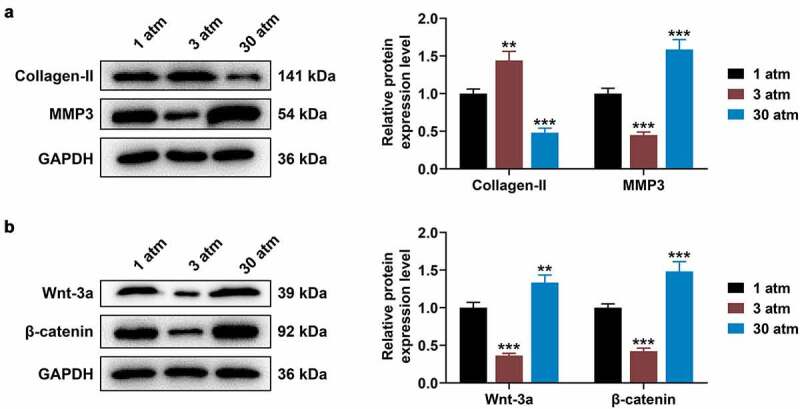
**High hydrostatic pressure (30 atm) inhibited Collagen-II expression, while promoting the levels of MMP3, Wnt-3a and β-catenin in HNPCs**. (A–B) HNPCs were collected, charged with cell medium in a plastic syringe and placed in the water-filled hydrostatic pressure-controlled vessel. Then cells were pressurized for 2 h under the hydrostatic pressure of 1, 3 and 30 atm. Next, the levels of Collagen-II, MMP3 (A), Wnt-3a and β-catenin (B) in treated HNPC were evaluated using Western blot, and GAPDH was used as the internal control. **P < 0.01, ***P < 0.001 vs. 1 atm (HNPCs: human nucleus pulposus cells, atm: atmospheres, MMP3: matrix metalloproteinase 3).

Previous results have shown that hydrostatic pressure regulated oxidative stress and chondrocyte metabolism in human osteoarthritic chondrocytes via modulating associated microRNA-mediated Wnt/β-catenin signaling pathway. In subsequent studies, the levels of Collagen-II, MMP3, Wnt-3a and β-catenin in treated HNPCs were evaluated using Western blot, the results of which displayed that 3 atm hydrostatic pressure treatment dramatically augmented Collagen-II expression, while diminishing MMP3 level in comparison to 1 atm hydrostatic pressure treatment (Figure 2A, P < 0.01). By contrast, in response to 30 atm hydrostatic pressure treatment, Collagen-II level tended to be decreased, but MMP3 expression tended to be increased (Figure 2A, P < 0.001). In addition, low expressions of Wnt-3a and β-catenin were associated with 3 atm hydrostatic pressure treatment (Figure 2B, P < 0.001), while high expressions of Wnt-3a and β-catenin were related to 30 atm hydrostatic pressure treatment, when compared to 1 atm hydrostatic pressure treatment (Figure 2B, P < 0.01). These data illustrated that high hydrostatic pressure (30 atm) may promote the apoptosis and inhibit the viability of HNPCs through regulating the Wnt/β-catenin signaling pathway.

### High hydrostatic pressure (30 atm) suppressed Collagen-II expression and promoted MMP3 expression in HNPC via promoting the Wnt/β-catenin pathway

**Figure 3.**
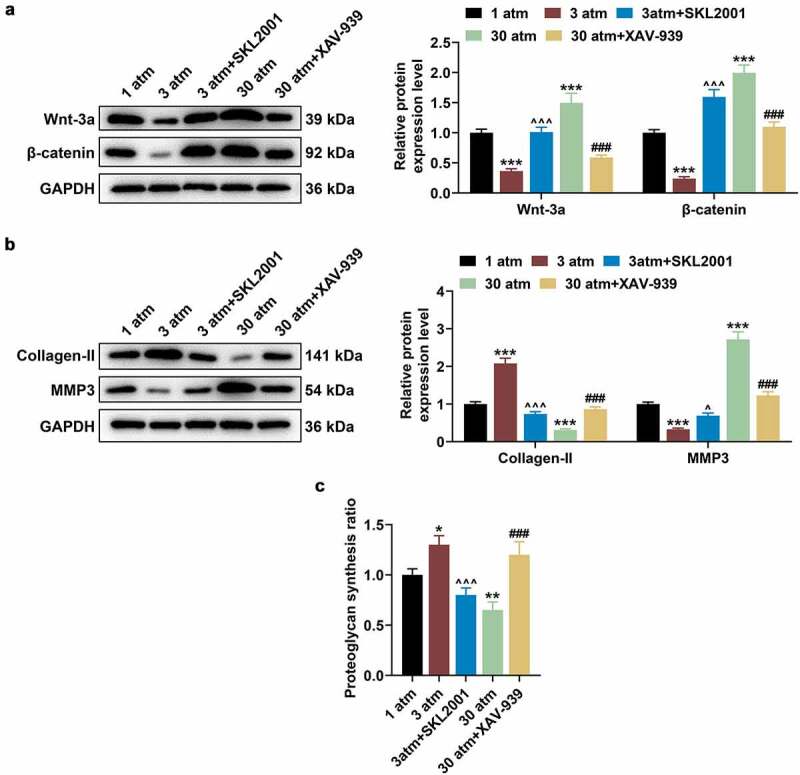
**High hydrostatic pressure (30 atm) inhibited the Collagen-II expression and promoted the MMP3 expression in HNPCs via activating the Wnt/β-catenin pathway**. (A–C) HNPCs were cultured in the cell medium containing SKL2001 or XAV-939, and cells were pressurized under the hydrostatic pressure of 3 or 30 atm for 2 h. Then, the levels of Wnt-3a, β-catenin (A), Collagen-II and MMP3 (B) in treated HNPC were evaluated using Western blot, and GAPDH was used as the internal control. The proteoglycan synthesis rate of HNPCs was evaluated using radioisotope incorporation assay (C). *P < 0.05, **P < 0.01, ***P < 0.001 vs. 1 atm; ^^^*P* < 0.05, ^^^^^*P* < 0.001 vs. 3 atm; ^###^*P* < 0.001 vs. 30 atm. (HNPCs: human nucleus pulposus cells, atm: atmospheres, MMP3: matrix metalloproteinase 3).

To further validate the above experimental results, HNPCs were cultured in the cell medium containing SKL2001 (40 μM) or XAV-939 (10 μM), after which cells were pressurized under the hydrostatic pressure of 3 or 30 atm for 2 h, and then collected for subsequent researches. After that, 3 atm hydrostatic pressure treatment brought about inhibited Wnt/β-catenin signaling pathway when compared with 1 atm hydrostatic pressure treatment, while SKL2001 reversed this inhibitory effect of 3 atm hydrostatic pressure treatment (Figure 3A; *P* < 0.001). Also, XAV-939 reversed the promoting effect of 30 atm hydrostatic pressure treatment on Wnt/β-catenin signaling pathway (Figure 3A; *P* < 0.001). Furthermore, SKL2001 counteracted the effect of 3 atm hydrostatic pressure treatment on promoting Collagen-II expression and inhibiting MMP3 expression in HNPCs (Figure 3B; *P* < 0.05), whilst XAV-939 reversed the effect of 30 atm hydrostatic pressure treatment on declining Collagen-II expression and increasing MMP3 expression in HNPCs (Figure 3B; *P* < 0.001). Similarly, 3 atm hydrostatic pressure treatment was uncovered to dramatically augment the proteoglycan synthesis ratio, while 30 atm hydrostatic pressuretreatment decreased the ratio (Figure 3C; *P* < 0.05). On this basis, SKL2001 reversed the promoting effect of 3 atm hydrostatic pressure treatment on proteoglycan synthesis ratio in HNPCs (Figure 3C; *P* < 0.001), whereas XAV-939 reversed the inhibitory effect of 30 atm hydrostatic pressure treatment on proteoglycan synthesis ratio in HNPCs (Figure 3C; *P* < 0.001). The results indicated that high hydrostatic pressure (30 atm) inhibited the Collagen-II expression and promoted the MMP3 expression in HNPCs via regulating the Wnt/β-catenin pathway.

## Discussion

The HNPC of the intervertebral disc plays a key role in resisting the load on the spine and intervertebral disc, and the main cause of intervertebral disc degeneration is usually considered to be a decrease in the number of HNPC [30–32]. One of the current discussions in intervertebral disc degeneration pertains to the effect and modulatory mechanism of hydrostatic pressure [33]. T Handa et al. have demonstrated that hydrostatic pressure influences intervertebral disc cell metabolism, 3 atm hydrostatic pressure may act as an anabolic factor for proteoglycan synthesis, and 1 atm or less or 30 atm or more hydrostatic pressure markedly inhibits proteoglycan synthesis [34]. Therefore, in this work, HNPCs were pressurized under the hydrostatic pressure of 1, 3 and 30 atm. Besides, a study has suggested that the nucleus pulposus cells undergo hydrostatic pressures and volume change during compression [34]. Previously, some researchers put forward that the nucleus pulposus cells are subjected to large hydrostatic pressure during compression, with little change in volume [34]. However, recent evidences have suggested that excessive hydrostatic pressure was associated with a reduction in volume of marrow stromal cells [35]. Additionally, intervertebral disc degeneration is accompanied by the apoptosis of HNPC [36]. In the current study, appropriate hydrostatic stimulation (3 atm) was discovered to improve the metabolism of HNPCs, while excessive hydrostatic pressure (30 atm) inhibited the viability and volume and promoted the apoptosis of HNPCs. However, the role of excessive hydrostatic pressure (30 atm) still needs further investigation.

As mentioned in the literature previously, the reduction of proteoglycans content in the nucleus pulposus is the starting process for the deterioration of the intervertebral disc [4]. A number of studies involving extracellular matrix and intervertebral disc degeneration have been reported, for example, Zhang et al. have indicated that melatonin promotes extracellular matrix remodeling and improves rat intervertebral disc degeneration [37]. The MMP family is instrumental during extracellular matrix metabolism, and MMP3, as a member of MMP family, is involved in the progression of intervertebral disc degeneration [38]. Furthermore, functional assays revealed that 17β-Estradiol suppresses intervertebral disc degeneration via decreasing MMP3 level and increasing Collagen-II expression in a rat model [39]. Collagen-II is one of the important constituent structures of cartilage and intervertebral disc, down-regulation of which induces apoptosis of HNPC [40]. In the present study, appropriate hydrostatic stimulation (3 atm) was confirmed to enhance the proteoglycan synthesis and the levels of extracellular matrix and Collagen-II but inhibit MMP3 levelin HNPCs, whereas excessive hydrostatic pressure (30 atm) generated the inverse impacts. The present study demonstrated that excessive hydrostatic pressure (30 atm) inhibited the viability and volume yet promoted the apoptosis of HNPC via inhibiting extracellular matrix and Collagen-II levels and elevating MMP3 expression.

Wnts are secreted glycoproteins that act as ligands to stimulate receptor-mediated signal transduction pathways, and activating Wnt pathway can regulate cellular behaviors [41]. The Wnt/beta-catenin pathway is well-known Wnt signaling pathway, and is linked to neurodegenerative diseases, cancers and bone density syndromes [41]. An increasing number of studies have reported that Wnt/β-catenin pathway participates in the progression of intervertebral disc degeneration [20]. In human osteoarthritic chondrocytes, hydrostatic pressure regulates oxidative stress via modulating the Wnt/β-catenin signaling pathway [42]. Silencing taurine up-regulated 1 protects HNPC from tumor necrosis factor-α-induced apoptosis, and also enhances cell proliferation by blocking Wnt/β-catenin pathway in HNPC [21]. Xie et al. have demonstrated that aquaporin 3 prevents lumbar intervertebral disc degeneration via the inhibition of Wnt/β-catenin signaling [28]. Similarly, Sun et al. have indicated that reduced Wnt/β-catenin signaling triggers the apoptosis of HNPC [43]. In this work, Wnt/β-catenin signaling pathway was verified to be inhibited when hydrostatic pressure was 3 atm, but was promoted when hydrostatic pressure was 30 atm. In the following tests, to fathom out the role of Wnt/β-catenin pathway in intervertebral disc degeneration, HNPCs were treated with SKL2001 (an agonist of the Wnt/β-catenin signaling pathway) or XAV-939 (an inhibitor of the Wnt/β-catenin pathway), and then pressurized under the hydrostatic pressure of 3 or 30 atm as previously described [44]. SKL2001 increases β-catenin responsive transcription via up-regulating the intracellular β-catenin protein level and inhibits the phosphorylation of β-catenin at residues Ser33/37/Thr41 and Ser45, which in turn disrupts Axin/β-catenin interaction [45]. XAV-939 selectively inhibits Wnt/β-catenin-mediated transcription by suppressing tankyrase1/2 [46] . This study found that SKL2001 reversed the inhibitory effect of hydrostatic pressure (3 atm) on Wnt/β-catenin signaling pathway, and XAV-939 reversed the promoting effect of high hydrostatic pressure (30 atm) on the pathway. Also, SKL2001 was confirmed to counteract the effect of hydrostatic pressure (3 atm), and XAV-939 reversed that of high hydrostatic pressure (30 atm) on proteoglycan synthesis as well as Collagen-II and MMP3 levels in HNPCs. The results evidenced that high hydrostatic pressure (30 atm) accelerated the apoptosis and inhibited the viability of HNPCs via promoting the Wnt/β-catenin pathway.

## Limitation

Although the current study revealed that high hydrostatic pressure (30 atm) modulated the physiological activity of HNPCs by regulating the Wnt/β-catenin pathway, an *in vivo* experiment still needs to be carried out to further verify the results of this experiment. In future studies, we plan to explore whether hydrostatic pressure regulates apoptosis of HNPCs by modulating associated microRNAs.

## Conclusion

To sum up, the research demonstrated that high hydrostatic pressure (30 atm) boosts the apoptosis and inhibits the viability of HNPCs via promoting the Wnt/β-catenin pathway. The results of this study provide a theoretical basis for the treatment of intervertebral disc degeneration.

## Highlights

- High hydrostatic pressure inhibits the viability yet promotes apoptosis of HNPCs.
- High hydrostatic pressure suppresses the extracellular matrix of HNPCs.
- High hydrostatic pressure impedes the proteoglycan synthesis of HNPCs.
- High hydrostatic pressure enhances the levels of Wnt-3a and β-catenin in HNPCs.
- XAV-939 reverses the effect of high hydrostatic pressure on Wnt-3a level in HNPCs.

## Supplementary Material

Supplemental Material

## Data Availability

The analyzed data sets generated during the study are available from the corresponding author on reasonable request.
