# Supplementary figures and images for: High hydrostatic pressure (30 atm) enhances the apoptosis and inhibits the proteoglycan synthesis and extracellular matrix level of human nucleus pulposus cells via promoting the Wnt/β-catenin pathway
Source: Bioengineered. 2022 Jan 31;13(2):3070–81. doi: 10.1080/21655979.2022.2025518 (PMC8974124; doi:10.1080/21655979.2022.2025518)

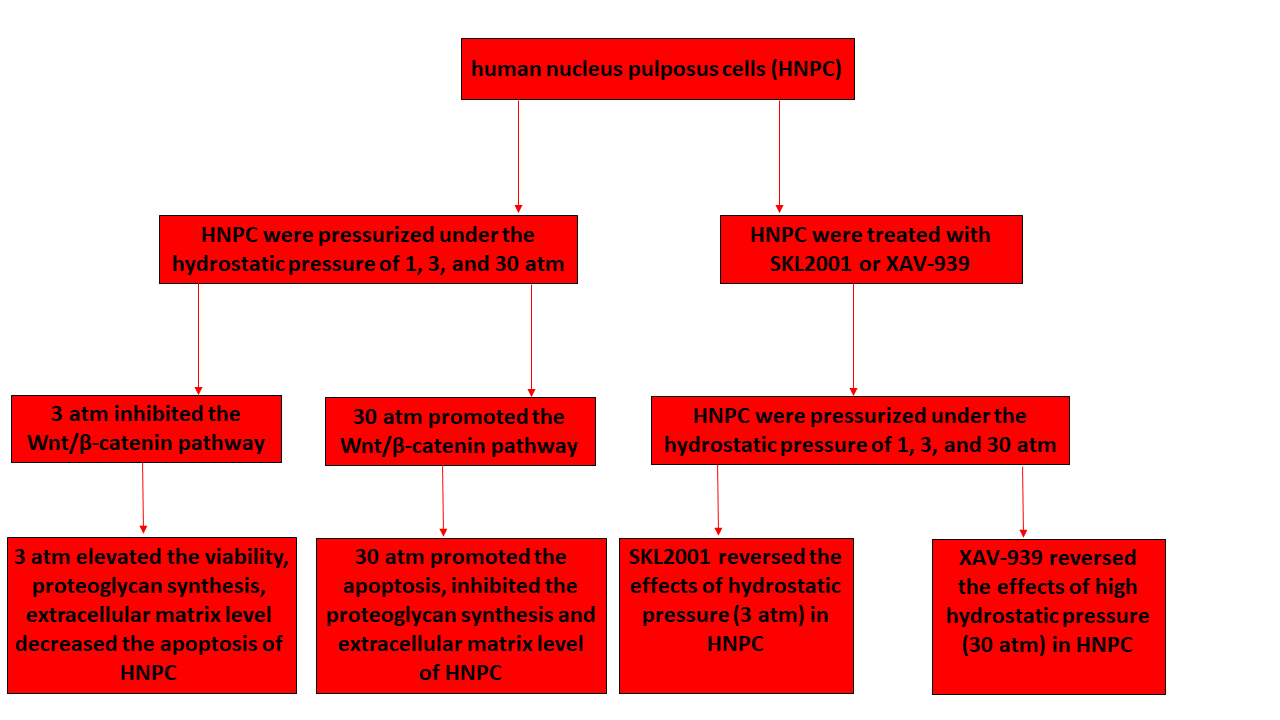

Supplement: Supplemental Material [file KBIE_A_2025518_SM1488.tif]
